# Focus on the nutritional intervention for healthy aging and human wellness based on the “environment-food-human” web

**DOI:** 10.3389/fnut.2024.1398916

**Published:** 2024-09-24

**Authors:** Hongkang Zhu, Xia Ou, He Qian, Norbu Dundrup

**Affiliations:** ^1^School of Food Science and Technology, Jiangnan University, Wuxi, Jiangsu, China; ^2^School of Medicine, University of Xizang Medicine, Lasa, China

**Keywords:** healthy aging, nutritional intervention, medicine and food homology, environment factors, one health

The aging of the global population is an impending health challenge and an opportunity of the 21st century; this trend is anticipated to accelerate over a quarter-century. The deterioration of the human physiology and psychology that accompany aging is a multifaceted phenomenon influenced by genetic and environmental factors. In the most severe cases, advanced age is a main risk factor for many chronic diseases and functional deficits in humans. Recently, López-Otín et al. (1) updated the theory of aging and defined 12 hallmarks of aging, reflecting the damages affecting the genome, telomeres, epigenome, proteome, and organelles. The hallmarks of the aging process are functionally interconnected. For example, there exists a strong correlation between nutritional dysregulation and aging-related diseases, such as high triglycerides, low high-density lipoprotein (HDL) cholesterol, high blood pressure, and type 2 diabetes. However, most determinants of the enormous variation in human lifespan can be attributed to environmental factors, including ecological environment and lifestyle factors (2). The aging hallmarks are interconnected to the eight hallmarks of health (3), achieving a closed-loop control for healthy aging through personalized nutritional interventions (4).

Diet, an environmental factor, substantially influences the course of human life and is increasingly recognized as a most practical target for anti-aging interventions (1, 5). Targeting nutrient sensors, intermittent fasting, and the ketogenic diet has become epidemic eating styles for anti-aging interventions and life extension (10%−20%) (6). Personalized nutritional interventions can include nicotinamide mononucleotide (NMN), vitamin D3, extracts of astragalus, mulberry leaf, and shiitake mushroom (7). Therefore, healthful nutrition has been acknowledged as a paradigm for prevention and intervention that promotes physical functioning and human wellness (8). A long history of traditional Chinese medicine (TCM) has documented a list of edible and medicinal foods and herbs with potential anti-aging and longevity properties. Polyacetylene was naturally isolated from carrots (*Daucus carota*) with age-related improvements in frailty, such as physical fitness, discomfort feelings, and respiratory function (9). Furthermore, Wang et al. (10) systematically screened Chinese herbal compounds and discovered that corylin (derived from *Psoralea corylifolia*) reduced the mortality of 102-week-old mice from 63.3% to 43.3% by inhibiting mTor1 phosphorylation. Currently, a massive range of TCMs with medicine and food homology characteristics, ranging from daily food to more exotic medicinal herbs, have been explored to delay the aging process and provide multiple health benefits. As an anti-aging strategy to prolong lifespan and prevent aging-related disorders, food-based nutritional intervention is triggering rising scientific interest and has been integrated into modern healthcare practices (11).

The concept of “homology medicine and food” originated in China but has transcended national boundaries, acquiring new significance in an aging society. TCM theory posits that medicine and food share the same origin and exhibit potential overlapping functions in the prevention and/or treatment of diverse health conditions. “Food is medicine” is undergoing a renaissance (12). Notably, it is generally ignored that there are noteworthy differences in the nutritional properties or functions of homologous medicine and food originating from different regions of production or growth. In recent decades, massive metabolite profiling of natural extracts has been extensively investigated and utilized with the advent of high-throughput technologies (13). Metabolomic studies have reported that geographical location and environmental factors could substantially influence metabolite profiling, thereby contributing to the disturbance of pharmacological activities (14). Therefore, the plateaus exhibit a greater prominence of the “trueborn area” where authentic medicinal herbs are cultivated, which appears to be crucial to their quality and nutritive value. For instance, eight kinds of “*Daodi*” Tibetan medicine (DTM) containing *Cordyceps sinensis* and *Brassica rapa* L. are listed by the Tibet Autonomous Region in 2024 (15). Most DTMs are established the anti-aging and lifespan-extensive resources in long-term practices. Cordycepin and other unique bioactive components could substantially inhibit cellular senescence and the senescence-associated secretory phenotype (SASP) factors (16).

Given the global importance of an aging society, the World Health Organization (WHO) has called for a comprehensive public-health response within an international legal framework (17). Aspects of the perspective of “one health” and the “environment-food-human” web contribute to the multidimensionality of the strategies of food-based dietary and nutritional interventions. Additional research is necessary to expand our knowledge of healthy aging and an extended lifespan, both of which are crucial for human health and wellness and satisfy the demands of the silver hair market.
